# An evaluation of ICD-11 PTSD and complex PTSD criteria in a sample of adult survivors of childhood institutional abuse

**DOI:** 10.3402/ejpt.v4i0.22608

**Published:** 2013-12-03

**Authors:** Matthias Knefel, Brigitte Lueger-Schuster

**Affiliations:** Faculty of Psychology, University of Vienna, Vienna, Austria

**Keywords:** ICD-11, posttraumatic stress disorder, complex PTSD, institutional abuse, gender

## Abstract

**Background:**

The WHO recently launched the proposal for the 11th version of the International Classification of Diseases (ICD-11) that also includes two diagnoses related to traumatic stress. In contrast to the Diagnostic and Statistical Manual of Mental Disorders, Fifth Edition (DSM-5), ICD-11 will probably, in addition to posttraumatic stress disorder (PTSD), also define a new diagnosis termed “complex posttraumatic stress disorder” (CPTSD).

**Objective:**

We aimed to apply the proposed ICD-11 criteria for PTSD and CPTSD and to compare their prevalence to the ICD-10 (International Classification of Diseases [10th revision]) PTSD prevalence. In addition, we compiled a list of symptoms for CPTSD based on subthreshold PTSD so as to include a wider group of individuals.

**Methods:**

To evaluate the appropriateness of the WHO ICD-11 proposal compared to the criteria of ICD-10, we applied the newly introduced criteria for PTSD and CPTSD deriving from the Posttraumatic Stress Disorder Checklist – Civilian Version (PCL-C) and the Brief Symptom Inventory (BSI) scales, to a sample of adult survivors (*N*=229) of childhood institutional abuse. We evaluated the construct validity of CPTSD using confirmatory factor analysis (CFA).

**Results:**

More individuals fulfilled the criteria for PTSD according to ICD-10 (52.8%) than the ICD-11 proposal (17% for PTSD only; 38.4% if combined with complex PTSD). The new version of PTSD neutralized the gender effects. The prevalence of CPTSD was 21.4%, and women had a significantly higher rate of CPTSD than men (40.4 and 15.8%, respectively). Those survivors who were diagnosed with CPTSD experienced institutional abuse for a longer time. CFA showed a strong model fit.

**Conclusion:**

CPTSD is a highly relevant classification for individuals with complex trauma history, but surprisingly, effects of gender were apparent. Further research should thus address gender effects.

The International Classification of Diseases (11th revision) (ICD-11) working group for mental disorders specifically associated with stress (Maercker et al., 2013) recently published a proposal for the ICD-11 criteria for posttraumatic stress disorder (PTSD). This proposal adopts a new diagnosis, termed “complex posttraumatic stress disorder” (CPTSD), that emphasizes clinical utility, in other words, consistency between diagnoses and clinicians’ mental health taxonomies (Cloitre, Garvert, Brewin, Bryant, & Maercker, 2013). CPTSD was initially proposed by Judith Herman (1992), who stated that “In contrast to the circumscribed traumatic event, prolonged, repeated trauma can occur only when the victim is in a state of captivity, unable to flee, and under control of the perpetrators” (p. 337). These complex traumatic events lead to more complex psychopathological symptoms which exceed normal PTSD. The Diagnostic and Statistical Manual of Mental Disorders, Fourth Edition (DSM-IV) working group suggested naming this syndrome “disorders of extreme stress not otherwise specified” (DESNOS; Friedman, Resick, Bryant, & Brewin, 2011). The DSM-IV field trial revealed that only 8% of individuals with DESNOS did not meet PTSD criteria as such; hence, no distinct diagnosis of CPTSD was implemented. However, a number of clinicians and researchers still claim that PTSD symptom clusters fail to assess clinically significant problems related to severe and protracted traumatic exposure, such as childhood sexual abuse or torture (de Jong, Komproe, Spinazzola, van der Kolk, & van Ommeren, 2005; Dickinson, deGruy, Dickinson, & Candib, 1998; Ford, 1999; Roth, Newman, Pelcovitz, van der Kolk, & Mandel, 1997; Sar, 2011; van der Kolk, Roth, Pelcovitz, Sunday, & Spinazzola, 2005). The symptom complexity following exposure to prolonged traumatic events includes deficits in regulating emotional stress (affect dysregulation), negative self-concept, interpersonal problems (Cloitre et al., 2013; Maercker et al., 2013), and dissociative symptoms (Sar, 2011). For cumulative childhood traumatic events in particular, many studies have confirmed these PTSD-exceeding symptoms (Cloitre et al., 2009; Courtois, 2004; Ford & Kidd, 1998; Herman, 1993; Zlotnick et al., 1996).

The recently published Diagnostic and Statistical Manual of Mental Disorders, Fifth Edition (DSM-5; American Psychiatric Association, 2013) moved PTSD from the previous chapter on “Anxiety Disorders” to include it in a chapter on “Trauma- and Stressor-related-Disorders.” The DSM-5 working group faced the same question as the ICD-11 working group, that is, whether or not a CPTSD diagnosis should be implemented. Therefore, the DSM-5 group reviewed the existing literature on CPTSD (Resick et al., 2012) and concluded that there is not enough empirical support for a distinct disorder, mainly because: (1) there is a lack of definitional consensus within and among researchers and practitioners; (2) there is a lack of reliable, valid measures; and (3) it is not yet clear if CPTSD depicts a separate construct rather than a more severe form of PTSD. However, the lively debate around CPTSD has continued despite the release of the DSM-5. The ICD-11 working group disagrees with the DSM-5 working group's conclusion and has countered that the adoption of CPTSD as a sibling diagnosis fosters clinical utility and accommodates the needs of clinicians. The proposed ICD-11 diagnosis comprises the ICD-11 core elements of PTSD, accompanied by affect disturbances, including disturbances relating to self and interpersonal relations. Cloitre et al. (2013) applied these new symptom clusters to a sample of adults who were exposed to different traumatic events, including single and prolonged traumatic events. In their latent profile analyses (LPA), they found three different symptom profiles: a group with generally low symptom distress, a group with high PTSD symptoms but low CPTSD symptoms, and a group with high PTSD symptoms and high CPTSD symptoms. The last group was significantly associated with more complex traumatic events, although some people who experienced prolonged traumatic events were allocated to the PTSD group by the LPA. Along with the ICD-11 working group, the authors conclude that CPTSD should be considered as an additional sibling diagnosis to PTSD with the traumatic stressor as a gate criterion, but trauma history is not determinative for the diagnosis (Cloitre et al., 2013; Maercker et al., 2012).

In contrast to their theoretical proposal, however, the measurement of PTSD and CPTSD seems to reflect a hierarchical rather than a horizontal relationship between PTSD and CPTSD in which CPTSD represents a more severe form of PTSD, requiring that the individual concerned fulfills the criteria for PTSD. Jonkman, Verlinden, Bolle, Boer, and Lindauer (2013) report that the likelihood of maltreated children meeting PTSD criteria after trauma seems to decrease when traumatization becomes more complex and more severe trauma-unrelated symptoms are reported. Hence, people with a history of complex traumatization might not report the diagnosis-relevant PTSD symptoms, and thus would not meet the criteria for CPTSD. This group would be at risk of not receiving a trauma-specific treatment. Furthermore, it has been proposed that an increasing number of different types of traumatic events, as is the case in institutional abuse (Wolfe, Jaffe, Jette, & Poisson, 2003), is related to an increasing number of different types of symptoms (Briere, Kaltman, & Green, 2008; van der Kolk et al., 2005). This seems to be especially true for cumulative childhood trauma (Cloitre et al., 2009).

In order to address this question, subthreshold PTSD could be considered as a pre-condition for CPTSD instead of full PTSD, which still includes the hierarchical relationship, but in a milder form than suggested by the ICD-11 working group. This approach might also be more consistent with the theoretical background emphasized by the ICD-11 working group. However, a detailed analysis of CPTSD based on different combinations of subthreshold PTSD symptoms is missing. A CPTSD diagnosis based on fewer PTSD symptoms might increase the chance of survivors of child maltreatment being diagnosed with a trauma-specific diagnosis and thus enhance their chance of receiving appropriate treatment, although other forms of treatment for survivors of childhood sexual abuse provide evidence for symptom reduction (Classen et al., 2011). Since PTSD and CPTSD are distinguished from other psychiatric disorders in that there is a known etiological component, survivors might also profit from the feeling of being less stigmatized compared to having other disorders.

A review of studies that implemented subthreshold PTSD diagnoses (e.g., Chandra, Satyanarayana, & Carey, 2009; Cukor, Wyka, Jayasinghe, & Difede, 2010; Schnurr et al., 2000) indicates that people with subthreshold PTSD exhibit less symptom severity and functional impairment than those with the full disorder, but significantly more than non-PTSD cohorts. Contributing to this debate, Friedman et al. (2011) proposed adding partial PTSD as a subtype of adjustment disorder, but this was not accepted by the DSM-5 working group (American Psychiatric Association, 2013). Although the evidence for subthreshold PTSD may not be sufficient, a good case can be made for its clinical utility. However, all previous studies followed the DSM criteria; no study was found that applied a model of subthreshold PTSD for ICD-10.

The purpose of our study is to address the following topics. Firstly, we aimed to compare the prevalences of PTSD according to ICD-10 and ICD-11 in a given sample of adult survivors of institutional child abuse. We further applied the proposed criteria for CPTSD and expected a high number of cases fulfilling these criteria, since the sample consisted of adult survivors of prolonged traumatic events in their childhood.

We further aimed to compare the duration of exposure to traumatic events between the groups of participants who were classified as having ICD-11 PTSD with those who were classified as having CPTSD. We hypothesized that those with CPTSD would have longer durations of exposure. All participants experienced at least one of the three types of violence (physical, sexual, emotional). We analyzed the pattern of the types of violence for the two groups (ICD-11 PTSD vs. CPTSD), assuming that the CPTSD group had experienced events involving a greater variety of types of violence. Current meta-analyses (Brewin, Andrews, & Valentine, 2000; Ozer, Best, Lipsey, & Weiss, 2003; Tolin & Foa, 2006) also refer to gender as a risk factor, and so we included gender as a factor in our analysis.

In order to address the hierarchical versus horizontal proposed definition of PTSD and CPTSD, we applied two different definitions for subthreshold PTSD as a precondition for CPTSD. This new approach might contribute to the ongoing debate, following the notion that subthreshold PTSD symptoms can also accompany CPTSD, to increase the chances of identifying an individual in need of a distinct treatment other than that for PTSD (Cloitre et al., 2011).

Finally, we aimed to explore the construct validity of CPTSD as a new diagnosis. Construct validity refers to the consistence of measurement of empirical data with the theoretical considerations of an underlying construct. We used confirmatory factor analysis (CFA) to test for construct validity, in terms of model fit, of CPTSD. Good model fit indicates that the understanding of the nature of CPTSD as it is proposed for the ICD-11 is consistent with the empirically measured data. The hypothesis in this case is that CPTSD consists of these four factors: PTSD, affect dysregulation symptoms, interpersonal problems, and negative self-concept. Cloitre et al. (2013) found a strong model fit for this factor structure of CPTSD. This result requires further validation and generalization to different samples. We therefore used confirmatory factor analytic procedures to address this topic.

## Study background

Several countries (e.g., USA, Ireland, and Germany) have been shaken by scandals within the Catholic Church or in institutions responsible for the care of children. These scandals publically revealed that thousands of children had been abused in institutions that claimed to care for them. The Catholic Church in Austria had to respond to survivors’ calls for justice and redress by establishing a victims’ protection commission. Victims from other institutions demanded justice as well, so that federal bodies set up local commissions to offer redress to these victims. We studied victims who had appealed to two different commissions. This study includes data from two groups (survivors of institutional abuse within the Catholic Church and within the federal organization for foster children) who had been in institutions from the 1960s to the 1990s. We analyzed documents from the victims’ redress process in order to qualify the nature and quantity of abuse experienced during their childhood.

## Methods

### Procedure

Participants in this study were individuals who had appealed to two different commissions. The specifics concerning their traumatic experiences were assessed by clinical psychologists and psychotherapists as part of the clearing process. These clearing documents included reports from clinical psychologists and psychotherapists who were deployed by the commissions, and which accurately covered the types of violence that were reported by the victims. The commission judged the reports for their credibility in order to redress the victims. At the time of our assessment, 795 individuals had appealed to the commission of the Catholic Church and 120 had appealed to the commission of the federal organization for foster children, giving a total of 915 individuals who were invited to enter our study. Of these, 448 of the Catholic Church sample and 58 of the foster children sample gave consent for analysis of their clearing documents. In addition, 234 and 46 individuals, respectively, for a total of 280 individuals, consented to participate in the questionnaire study.

Participants were invited to come to the Faculty of Psychology in the University of Vienna for the assessment, or were offered support via phone to fill in the questionnaires at home and post them back. For further, detailed information on the background and context, see Lueger-Schuster et al. (2013).

### Participants

Data from 229 individuals (25.03% of 915) were available for analysis; 47 did not return the questionnaires, 22 withdrew their consent, and two were excluded because of too many missing values. There were 177 men (77.3%) and 52 women (22.7%). The average age of the sample was 55.8 years (SD 9.8); the youngest participant was 24 years old and the oldest 80 years old. The majority were married/cohabiting (62.9%), 16.6% were single, 14.8% were divorced, and the remaining 5.7% were either widowed or reported no information on their civil status. The civil status of the sample was representative for the Austrian population (Lueger-Schuster et al., 2013). Information on highest education obtained was available for 223 individuals: 20.2% had completed compulsory school or less, 50.7% had completed an apprenticeship, 18.8% had attended high school (A-level-equivalent), and 10.3% had a university or college degree.

All participants experienced institutional child abuse in the form of physical abuse (67.7%), emotional abuse (82.5%), or sexual abuse (69.9%). In all, 13.5% were exposed to traumatic events of one type of violence, 53.3% to traumatic events of two types of violence, and 33.6% were exposed to violent traumatic events of all three types of violence during childhood. There were no gender-related differences in the pattern of experienced traumatic events: men and women did not differ significantly in their experiences of certain types of violence (physical, sexual, emotional) (all df = 1, all χ^2^ ≤ 2, all *p*≥0.16) or in the combination of the three types of violence (one to three) (df = 2, χ^2^ = 3.2, *p*=0.2).

### Measures

To assess the ICD-10 PTSD, ICD-11 PTSD, and CPTSD criteria, we used two self-rated questionnaires: The Posttraumatic Stress Disorder Checklist – Civilian Version (PCL-C; Weathers, Litz, Herman, Huska, & Keane, 1991) and the Brief Symptom Inventory (BSI; Derogatis & Melisaratos, 1983). The PCL-C examines 17 symptoms of PTSD based on the DSM-IV (American Psychiatric Association, 2000). It has good psychometric properties to reliably detect PTSD (Weathers et al., 1991). On a five-point Likert scale (1 = “none” to 5 = “very”), participants rated the symptoms experienced in the past 4 weeks. A symptom is classified as present if participants rate it with 3 (“moderately”) or higher. The German translation of the PCL-C (Teegen, 1997) was used in this study. The BSI is a self-report measure of clinically relevant psychological symptoms with empirically evaluated reliability and validity (Derogatis & Melisaratos, 1983; Franke & Derogatis, 2000). Participants rated 53 items regarding their symptom distress on a five-point Likert scale (0 = “not at all” to 4 = “extremely”). The same cut-off approach as for the PCL-C was administered, thus a rating of 2 (“moderately”) or higher was counted as a present symptom.

For subthreshold PTSD, we developed two different models. Since the ICD-11 proposal asks only for one symptom within each criterion, some of the commonly used versions were not applicable (e.g., one symptom of B, one symptom of C, and one symptom of D). We tested two other models of subthreshold PTSD in accordance with the ICD-11 compilation of symptoms. Model 1 (SUB1 ICD-11) includes re-experiencing and avoidance or sense of threat, and model 2 (SUB2 ICD-11) requires a combination of any two criteria. Thus, both models also include individuals with the full PTSD diagnosis. In detail, SUB1 and SUB2 differ only insofar as SUB2 also includes those who experience the avoidance and the sense of threat criteria, but not the re-experiencing criterion. Therefore, the prevalence of SUB2 has to be at least equivalent to or higher than the prevalence of SUB1. To compute subthreshold CPTSD, we added the symptoms of ICD-11 as defined by Cloitre et al. (2013) to each of the models, and thus no changes in the proposed symptoms for the CPTSD part were made, only for the PTSD part.

### ICD-10 PTSD, ICD-11 PTSD, and CPTSD

The algorithm we used to assess ICD-10 PTSD was proposed by the U.S. Department of Veterans Affairs (2013). For ICD-11 PTSD and CPTSD, we followed the criteria applied by Cloitre et al. (2013). Table 1 displays a comprehensive overview of the combinations of the symptoms.

**Table 1:** Overview on and comparison of (complex) PTSD symptoms according to ICD-10 and ICD-11

			ICD-10		ICD-11			
				
Symptom cluster	Symptom	Corresponding item	PTSD		PTSD		CPTSD	
Re-experiencing	Intrusive flashbacks, vivid memories, or recurring dreams	PCL-C item 1: Repeated, disturbing memories, thoughts, or images of a stressful experience from the past?	*	At least one symptom				
		PCL-C item 2: Repeated, disturbing dreams of a stressful experience from the past?	*		*	At least one symptom	*	At least one symptom
		PCL-C item 3: Suddenly acting or feeling as if a stressful experience were happening again (as if you were reliving it)?	*		*		*	
	Experiencing distress when reminded of the stressor	PCL-C item 4: Feeling very upset when something reminded you of a stressful experience from the past?	*					
		PCL-C item 5: Having physical reactions (e.g., heart pounding, trouble breathing, or sweating) when something reminded you of a stressful experience from the past?	*					
Avoidance	Internal avoidance	PCL-C item 6: Avoid thinking about or talking about a stressful experience from the past or avoid having feelings related to it?	*	At least one symptom	*	At least one symptom	*	At least one symptom
	External avoidance	PCL-C item 7: Avoid activities or situations because they remind you of a stressful experience from the past?	*		*		*	
Hyperarousal	Inability to recall	PCL-C item 8: Trouble remembering important parts of a stressful experience from the past?	*	Inability to recall or at least two other symptoms				
	Sleep problems	PCL-C item 13: Trouble falling or staying asleep?	*					
	Irritability	PCL-C item 14: Feeling irritable or having angry outbursts?	*					
	Concentration problems	PCL-C item 15: Having difficulty concentrating?	*					
	Hypervigilance	PCL-C item 16: Being “super alert” or watchful on guard?	*		*	At least one symptom	*	At least one symptom
	Exaggerated startle response	PCL-C item 17: Feeling jumpy or easily startled?	*		*		*	
Affect dysregulation		BSI item 13: Temper outbursts that you could not control					*	At least one symptom
		BSI item 20: Your feelings easily hurt					*	
Negative self-concept		BSI item 50: Feelings of worthlessness					*	At least one symptom
		BSI item 52: Feelings of guilt					*	
Interpersonal problems		BSI item 44: Never feeling close to another person					*	At least one symptom
		PCL-C item 10: Feeling distant or cut off from other people?					*	

* Indicates possible symptoms.

BSI, Brief Symptom Inventory; CPTSD, complex posttraumatic stress disorder; ICD-10, International Classification of Diseases (10th revision); ICD-11, International Classification of Diseases (11th revision); PCL-C, Posttraumatic Stress Disorder Checklist – Civilian Version; PTSD, posttraumatic stress disorder.

### Confirmatory factor analysis

We aimed to analyze the CPTSD structure, which consists of four factors: PTSD, affect dysregulation symptoms, interpersonal problems, and negative self-concept. In order to compare our results with those recently published by Cloitre et al. (2013), we used the same statistical procedure: prior to computation of the CFA, we standardized all included items. In the CFAs, we used correlation/covariance matrices based on Pearson's correlations. To assess normality assumptions for maximum likelihood parameter estimation (MLE), we calculated skewness and kurtosis and considered values of less than 2 and 7, respectively, as satisfying the assumption of normality. Residual covariance was allowed for the measurement pairs within each of the three PTSD factors and it was fixed to 0 for all other residuals. The factor loadings of the 3×2 items for each of the three factors assessing self-regulation problems were fixed to 1, to make the model identified. To assess model fit, we used the following indices: comparative fit index (CFI), Tucker-Lewis index (TLI), and root mean-square error of approximation (RMSEA). We followed the suggested benchmarks as indicators for good model fit: CFI >0.95, TLI >0.95, and RMSEA <0.06 (Schreiber, Nora, Stage, Barlow, & King, 2006).

## Results

### Prevalence

The prevalences of all ICD-11 PTSD and CPTSD symptoms are presented in Table 2. The most prevalent PTSD symptom was internal avoidance, which was reported by 55.5% of the total sample. Being easily hurt, which is one of the two affect dysregulation symptoms, was the most prevalent CPTSD symptom and was reported by 55.5% of the sample. Within the PTSD symptom clusters, there was a gender difference only for arousal, with higher rates for women. All three CPTSD symptom clusters were reported significantly more often by women.

**Table 2:** Gender-specific prevalence of ICD-11 PTSD and CPTSD symptoms

	Total% (*N*=229)	Men% (*N*=177)	Women% (*N*=52)
Re-experiencing	59.0	55.9	69.2
Repeated, disturbing dreams of a stressful experience from the past?	52.4	49.7	61.5
Suddenly acting or feeling as if a stressful experience were happening again (as if you were reliving it)?	40.2	36.2	53.8
Avoidance	62.4	58.2	76.9
Avoid thinking about or talking about a stressful experience from the past or avoid having feelings related to it?	55.5	51.4	69.2
Avoid activities or situations because they remind you of a stressful experience from the past?	45.4	41.2	59.6
Arousal	57.6	53.1	73.1*
Being “super alert” or watchful on guard?	50.2	46.3	63.5
Feeling jumpy or easily startled?	41.5	36.2	59.6*
Affect dysregulation	62.9	58.8	76.9*
Temper outbursts that you could not control	39.5	38.6^a^	42.3
Your feelings easily hurt	55.5	50.6^a^	72.5^c,^ *
Negative self-concept	46.7	41.8	63.5*
Feelings of worthlessness	40.1	34.3^b^	59.6*
Feelings of guilt	31.1	27.7	43.1^c^
Interpersonal problems	57.6	53.1	73.1*
Never feeling close to another person	42.1	39.0	52.9^c^
Feeling distant or cut off from other people?	43.7	37.9	63.5*
At least one symptom in each of the three factors assessing self-regulation problems	33.2	28.2	50.0*

* Significant difference (*p*<0.01) with higher rates for women.

a *N*=176

b *N*=175

c *N*=51.

CPTSD, complex posttraumatic stress disorder; ICD-11, International Classification of Diseases (11th revision); PTSD, posttraumatic stress disorder.

The prevalences of ICD-10 PTSD, ICD-11 PTSD, and CPTSD are given in Table 3. The rate of PTSD decreased from ICD-10 to ICD-11, declining from 52.8 to 38.4% if those with CPTSD are included. Criteria for CPTSD were fulfilled by 21.4% of the sample. Separately calculated prevalences for men and women showed that: (1) significantly more women than men (*p*<0.01) fulfilled ICD-10 PTSD criteria; (2) the prevalence of ICD-11 PTSD (if those with CPTSD were excluded) did not differ between women and men; and (3) significantly more women than men (*p*<0.01) fulfilled CPTSD criteria (see Table 3). A group of 24.0% showed SUB1 PTSD and a further group of 24.0% met the criteria for SUB1 CPTSD (Table 3). Again, women showed significantly higher rates than men only for SUB1 CPTSD (*p*<0.01; see Table 3). SUB2 PTSD was similarly distributed. The prevalence for both SUB2 PTSD and SUB2 CPTSD was 27.1%; women showed a higher rate of SUB2 CPTSD, but not of SUB2 PTSD. Interestingly, the latter was more prevalent in men, but the difference was not statistically significant.

**Table 3:** Prevalence of PTSD, CPTSD, and SUB CPTSD

	ICD-10	ICD-11			SUB1 ICD-11^a^			SUB2 ICD-11^b^		
				
	PTSD	PTSD	CPTSD	Both	SUB1 PTSD	SUB1 CPTSD	Both	SUB2 PTSD	SUB2 CPTSD	Both
Total	121 (52.8)	39 (17.0)	49 (21.4)	88 (38.4)	55 (24.0)	55 (24.0)	110 (48.0)	62 (27.1)	62 (27.1)	124 (54.2)
Men	84 (47.4)	30 (16.9)	28 (15.8)	58 (32.8)	44 (24.9)	32 (18.1)	76 (42.9)	51 (28.8)	39 (22.0)	90 (50.8)
Women	37 (71.2)*	9 (17.3)	21 (40.4)*	30 (57.7)*	11 (21.2)	23 (44.2)*	34 (65.4)*	11 (21.2)	23 (44.2)*	34 (65.4)

* Significant difference (*p*<0.01) with higher rates for women.

a SUB1 ICD-11 PTSD symptoms consist of at least B and (C or D), with no changes in the proposed additional symptoms for CPTSD;

b SUB2 ICD-11 PTSD symptoms consist of at least any 2 criteria, with no changes in the proposed additional symptoms for CPTSD.

CPTSD, complex posttraumatic stress disorder; ICD-10, International Classification of Diseases (10th revision); ICD-11, International Classification of Diseases (11th revision); PTSD, posttraumatic stress disorder; SUB subthreshold.

About one third of participants (76 persons) demonstrated the self-regulation problems that are required for a CPTSD diagnosis (see Table 2). However, not all of them met the criteria for ICD-11 PTSD and so only 64.5% of this group (49 persons) were diagnosed with CPTSD. When the subthreshold diagnoses for ICD-11 PTSD were applied, this rate increased to 72.3% (55 persons) for SUB1 PTSD and to 81.6% (62 persons) for SUB2 PTSD (Table 3).

To test for differences in duration of exposure to traumatic events, we used an ANOVA and planned contrasts. Although there was no main effect (*F*(2,221) = 48.46, *p*=0.085, partial *η^2^ =* 0.022), the more powerful planned contrasts revealed that the CPTSD group had longer duration of exposure in comparison to the ICD-11 PTSD group (*t*(221) = 2.23, *p*=0.027, *d*=0.62), while the group without ICD-11 PTSD did not differ significantly from the other two groups (*t*(221) = 0.31, *p*=0.759).

No differences in the combination of types of violence (one to three types) were found between the sample without ICD-11 PTSD, with ICD-11 PTSD, and with CPTSD (χ^2^ = 4.4, df = 4, *p*=0.36).

### Confirmatory factor analysis

The normality assumption tests were satisfied, as the skewness and kurtosis values were smaller than the critical values (2 and 7, respectively), so we used MLE. The CFA model of CPTSD showed a very good model fit: CFI = 0.98, TLI = 0.97, RMSEA = 0.05; 90% confidence interval (CI): 0.03, 0.07. The correlations for PTSD and the three CPTSD factors varied from *r*=0.45 to *r*=0.49, and the correlations of the three CPTSD factors varied from *r*=0.42 to *r*=0.52 (Table 4).

**Table 4:** Correlation matrix of the CPTSD symptom clusters

	1	2	3
1 PTSD			
2 Affect dysregulation	0.45		
3 Negative self-concept	0.45	0.45	
4 Interpersonal problems	0.49	0.42	0.52

CPTSD, complex posttraumatic stress disorder; PTSD, posttraumatic stress disorder.

## Discussion

In a sample of adult survivors of institutional child abuse (*N*=229), we used data from the PCL-C and the BSI and combined them with the analysis of documents originating from a process of redress from two different victim protection commissions in Austria. The history of child abuse was intensively documented in these records. The institutional background was the Catholic Church and some federal organizations located close to Vienna. All participants experienced exposure to severe multiple and complex traumatic events in their childhood (Lueger-Schuster et al., 2013). We aimed to look into the gender-specific prevalence and validity of PTSD and CPTSD symptoms and diagnoses as compiled for ICD-11. Additionally, we analyzed two models of subthreshold PTSD as a basis for CPTSD to include those individuals who do not fulfill the criteria for PTSD but report the CPTSD-specific symptoms as suggested for ICD-11.

### PTSD and CPTSD symptoms and diagnoses

A high rate of 52.8% ICD-10 PTSD was identified, based on the classic core symptoms. Although ICD-11 includes only six PTSD symptoms, which might lead to the premature expectation of higher prevalences, the ICD-11 PTSD rate is evidently lower (38.4%, including those who also fulfill the CPTSD criteria). This rate was similar to those in other studies that investigated samples with a complex traumatic exposure (Kessler, Sonnega, Bromet, Hughes, & Nelson, 1995; Tolin & Foa, 2006). Considering the lower threshold for PTSD in ICD-10 compared to DSM-IV (Maercker et al., 2013), the reduction in the PTSD prevalence from ICD-10 to ICD-11 could reflect a convergence of ICD and DSM. Further research comparing PTSD rates identified by DSM-5 and ICD-11 could investigate this possible convergence. When those persons who in future would be diagnosed with CPTSD are excluded, the ICD-11 prevalence of PTSD drops to 17.0%, resulting in a rate of 21.4% for the total CPTSD sample. This concurs with our hypothesis that our complex traumatized sample exhibits higher CPTSD rates, and is consistent with the theoretical background of CPTSD. Also consistent with the theoretical foundations of CPTSD as a disorder following prolonged traumatic events, we found that those who were diagnosed with CPTSD were exposed for significantly longer to traumatizing situations in institutional or foster care settings. Nevertheless, this finding has to be treated with caution, because no main effect of the analysis was found and only the more powerful planned contrast analysis revealed the expected group differences. Still, many people with prolonged traumatic experiences report PTSD symptoms rather than CPTSD symptoms. The ICD-11 working group (Cloitre et al., 2013; Maercker et al., 2013) depicts the event-criterion as a risk factor but not as a compelling risk factor. The so-called “gate” criterion allows for both PTSD and CPTSD. The results of the present study support this assumption.

CFA is used to test for construct validity and thus is applied to predefined theoretical models. We wanted to test the concordance between the proposed theoretical construct of CPTSD and the empirical results. The good model fit, even slightly superior to the fit Cloitre et al. (2013) found in their analyses, additionally supports the construct validity of the ICD-11 proposal. This finding might relate to our sample that consists of adults with a complex childhood trauma history.

### Gender-specific evaluation

The gender-specific evaluation for ICD-10 PTSD shows the typically observed higher prevalence of PTSD in women (Brewin et al., 2000; Ozer et al., 2003; Tolin & Foa, 2006). The prevalence of ICD-11 PTSD and CPTSD together shows the same unbalanced distribution. Interestingly, this imbalance disappears in ICD-11 PTSD, and seems to shift towards CPTSD: no gender difference was observed in ICD-11 PTSD, but it was observed in CPTSD. Experiencing CPTSD symptoms such as affect dysregulation, negative self-concept, interpersonal problems, and higher arousal, seems to be more representative of females. The question that arises is: where does this gender bias come from? Former studies investigating adult survivors of child abuse refer mostly to female samples (Briere, & Jordan, 2004; Cloitre, Miranda, Stovall-McClough, & Han, 2005; Cloitre et al., 2009; Widom, 1999; Widom, Czaja, Bentley, & Johnson, 2012; Widom, DuMont, & Czaja, 2007), and the early drafts of DESNOS also came from female-dominated samples or clinical experiences with female rape victims (Cloitre et al., 2005; Cloitre, Scarvalone, & Difede, 1997; Cloitre et al., 2009; Courtois, 2004; Herman, 1992; Mechanic, Uhlmansiek, Weaver, & Resick, 2000; Roth et al., 1997; Taft & Hegarty, 2010). Only a few studies have looked into the symptoms of male prisoners of war (Dikel, Engdahl, & Eberly, 2005), torture survivors (de Jong et al., 2005), or survivors of other intentional traumatic exposure (Andrews, Brewin, & Rose, 2003; Santiago et al., 2013).

The suggestion of Freyd and colleagues (as cited in Friedman, Keane, & Resick, 2007, p. 216) that gender differences are a side effect of the experience of different types of traumatic events resulting in differences in psychopathology, is not supported in our study because men and women experienced the same types of abuse (Lueger-Schuster et al., 2013).

Does this symptom representation border on a stereotypical role definition that considers women generally weaker and more emotionally focused than men? Costa, Terracciano, and McCrae (2001) found very robust empirical support for gender stereotypes across cultures: women report higher values in neuroticism, agreeableness, warmth, and openness to feelings, while men scored higher in assertiveness and openness to ideas. Moreover, Kimerling, Ouimette, and Weitlauf (2007) hint at the issue that social roles may moderate the impact of posttrauma responses (e.g., helplessness and emotional distress), as posttrauma cognitions are more consonant for women (Baker et al., 2005). Based on these findings, a female stereotype of posttrauma symptoms might also include lamenting, irritability or nervousness, a tendency to be reluctant to confront issues, and problems concerning self-concept. Miller and Resick (2007) compared their results on adult trauma survivors to those of Miller, Greif, and Smith (2003) and Miller, Kaloupek, Dillon, and Keane (2004) and found that men tend to display externalizing symptoms of CPTSD, while women endorse internalizing symptoms of CPTSD. The internalizing subtype of CPTSD includes symptoms such as hopelessness, shame, high detachment, and feelings of ineffectiveness; in contrast, the externalizing subtype of CPTSD includes symptoms such as self-destructive and impulsive behavior, and hostility. The present gender effect in this study might be attributed to the set of symptoms that was used to measure CPTSD (except for temper outbursts), which rather reflects the internalizing than the externalizing subtype. However, further research should focus on gender-specific aspects in CPTSD in order to better understand the underlying mechanisms.

### Subthreshold PTSD and Subthreshold CPTSD

We stated that the proposed criteria for CPTSD require all PTSD symptoms, and thus PTSD and CPTSD can be seen rather as vertically dependent disorders than as sibling disorders. The theoretical considerations lead to the conclusion that CPTSD manifests in different, albeit overlapping, symptoms to those of PTSD. Nearly one third of the sample reported symptoms included in all three CPTSD-specific criteria, but only 21.4% were diagnosed with CPTSD, leaving many people without a trauma-specific diagnosis because they did not fulfill all necessary PTSD criteria. We cannot conclude from the BSI items whether this group might qualify for borderline personality disorder (BPD) or depression; having been exposed to severe maltreatment during their childhood was considered as an etiological agent for the complex trauma symptomatology. According to Resick et al. (2012), trauma exposure should show a stronger magnitude with complex trauma symptoms than with BPD. McLean and Gallop (2003) specify that if the diagnosis of CPTSD can be given for cases with childhood sexual abuse, the axis II diagnosis of BPD could be subsumed under the construct of CPTSD. To cover more of those individuals with CPTSD-specific symptoms who did not meet all PTSD criteria, we applied two definitions of subthreshold PTSD as a precondition for CPTSD. Clearly, this resulted in higher prevalences than the ICD-11 diagnostic criteria (PTSD and CPTSD together: 48.0 and 54.2% for subthreshold PTSD 1 and subthreshold PTSD 2, respectively), but considering the ICD-10 PTSD prevalence of 52.8%, these rates seemed to be in an acceptable range. The number of those who missed a diagnosis decreased from ICD-11 PTSD to subthreshold PTSD 1 to subthreshold PTSD 2. Considering that the entire sample of the present study was exposed to prolonged interpersonal traumatic events, a prevalence of 27.1% for CPTSD (with subthreshold PTSD 2 as a condition) appears to be appropriate and not over-diagnosed. The total prevalence of CPTSD increases from 21.4 to 27.1%, which is an increase of only 5.7%. Still, the proportion of individuals who fulfilled the CPTSD-specific symptoms without the PTSD-specific symptoms, rises from 64.5% (49 of 76 persons) to 72.3% (55 of 76 persons) for subthreshold PTSD 1 and to 81.6% (62 of 76 persons) for subthreshold PTSD 2. Subthreshold PTSD 2 also includes individuals who do not report re-experience symptoms, thus the question arises: is it still PTSD if there is no re-experiencing?^1^ For clinicians, a verified distinction between PTSD and CPTSD as suggested for ICD-11 would be helpful. If subthreshold PTSD symptoms can also accompany CPTSD, the chances of identifying an individual in need of a distinct treatment other than that for PTSD (Cloitre et al., 2011) increases. This approach seems very promising to us and could be considered in further reflections of the ICD-11 working group.

### Limitations

A limitation of our study is the very specific sample, which is male-dominated and consists of adult survivors of institutional child abuse. A long duration between the traumatization and the disclosure might bias the memories relating to the abusive situations. Generally, people tend to under-report traumatic incidents (Edwards, Holden, Felitti, & Anda, 2003). The most important limitation concerns the measurement and diagnostics: the measurement of a new diagnosis is at this early stage not yet validated, but we applied the same procedure as Cloitre et al. (2013) in order to compare our results. However, we used only a few items of screening instruments to measure CPTSD, which cannot compete with structured clinical interviews. The BSI might be limited in its ability to measure affect dysregulation and negative self-concept, and the present study did not implement an impairment measure, as required for an ICD-11 diagnosis. Additionally, it is possible that at least some people had personality disorders (Cohen, Brown, & Smailes, 2001), which we did not measure. However, all of them were exposed to complex trauma, so the diagnosis of BPD might be of less relevance (McLean & Gallop, 2003).

Furthermore, we do not know how many individuals in our sample have received treatment and, if so, what type of treatment.

## Conclusion

In ICD-11, PTSD will be redefined and CPTSD will probably be introduced. From the present study on adult survivors of complex interpersonal child abuse, we conclude that CPTSD seems to be an important clinically relevant diagnosis, which should be considered in ICD-11 and in treatment research (Cloitre et al., 2011). It appears that CPTSD is a female-dominated disorder. Further replication of this result is needed and future research should address the specific mechanisms underlying a potential gender bias in CPTSD. Reliable and valid instruments for screening and clinical diagnostics based on the proposed criteria should be developed and tested.

The structure of CPTSD repeatedly shows good construct validity, although further research should address the question of gender-specific construct validity. The question whether CPTSD represents a distinct or a sibling disorder is not yet clear; nevertheless, the present results suggest that both approaches are promising. We therefore conclude that in addition to the CPTSD-specific symptoms, the inclusion of some PTSD symptoms might be the best way to define CPTSD.

## Footnotes

1 We thank the editor for the helpful comment.
